# Magnet-assisted traction method helps to reduce the difficulty of esophageal endoscopic submucosal dissection

**DOI:** 10.1055/a-2186-5029

**Published:** 2023-11-29

**Authors:** Yuan Gao, Wei Liu, Liansong Ye, Jiang Du, Jia Xie, Qiongying Zhang, Bing Hu

**Affiliations:** Department of Gastroenterology and Hepatology, West China Hospital, Sichuan University, China

Endoscopic submucosal dissection (ESD) is the main treatment of early esophageal cancers and precancerous lesions. However, esophageal ESD is extremely demanding technically because sometimes exposure of the submucosal layer is challenging, which increases the difficulty of the operation and the risk of perforation 1 2 . In this study, we used a magnet-assisted traction method to reduce the difficulty and increase the safety of esophageal ESD.

A 55-year-old woman underwent a routine gastroscopy examination. It was demonstrated that a suspected lesion was located in the anterior wall of the middle esophagus ( Fig. 1 ). Biopsy pathology revealed a low grade intraepithelial neoplasia (LGIN). The patient strongly requested endoscopic resection because she had a family history of esophageal cancer. Then, ESD was planned. However, after injection and incision, exposure of the submucosal layer was not satisfactory. Therefore, we decided to apply this magnet-assisted traction method. First, we fixed a magnetic bead with attached thread to the edge of the lesion by using an endoclip. After that, we used another external powerful magnet in the middle of the two scapulae to apply traction ( Fig. 2 ). Meanwhile, we adjusted the direction of traction by changing the position of the magnet outside the body. Thereafter, a clear cutting line was exposed so that submucosal dissection could be performed smoothly without any adverse events ( Fig. 3 , Video 1 ). Postoperative pathology confirmed LGIN with negative margins.

**Fig. 1:**
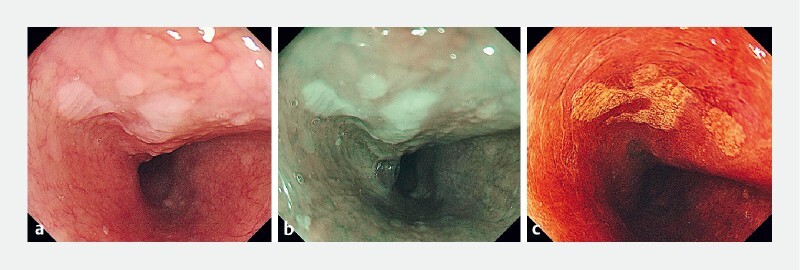
Preoperative endoscopic examination of the esophageal lesion, 21–24 cm from incisor teeth. **a** White light appearance. **b** Narrow-band imaging. **c** Iodine staining.

**Fig. 2:**
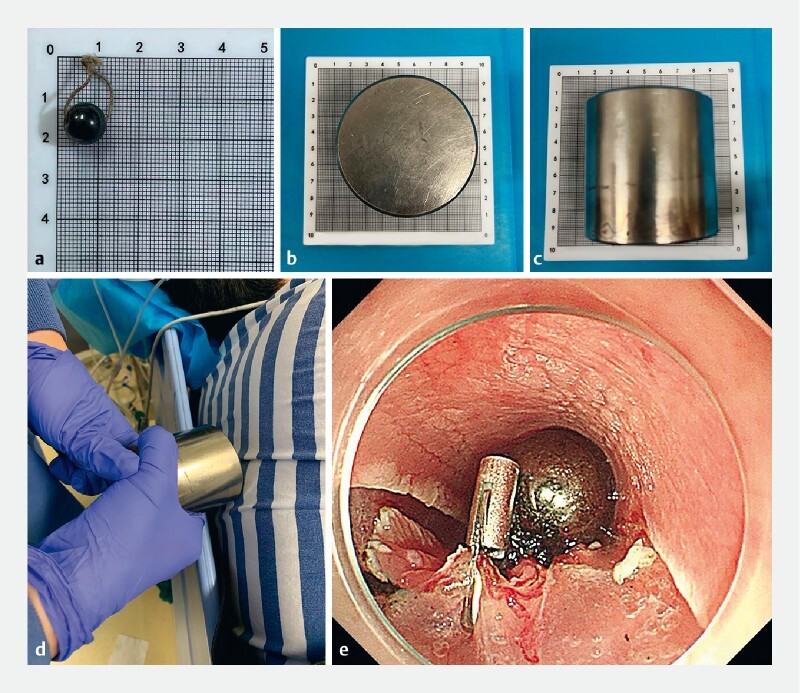
The magnet system. **a** The internal magnetic bead. **b, c** The external magnet. **d** The external magnet (placed in the middle of the two scapulae). **e** The internal magnetic bead (fixed to the edge of the lesion).

**Fig. 3:**
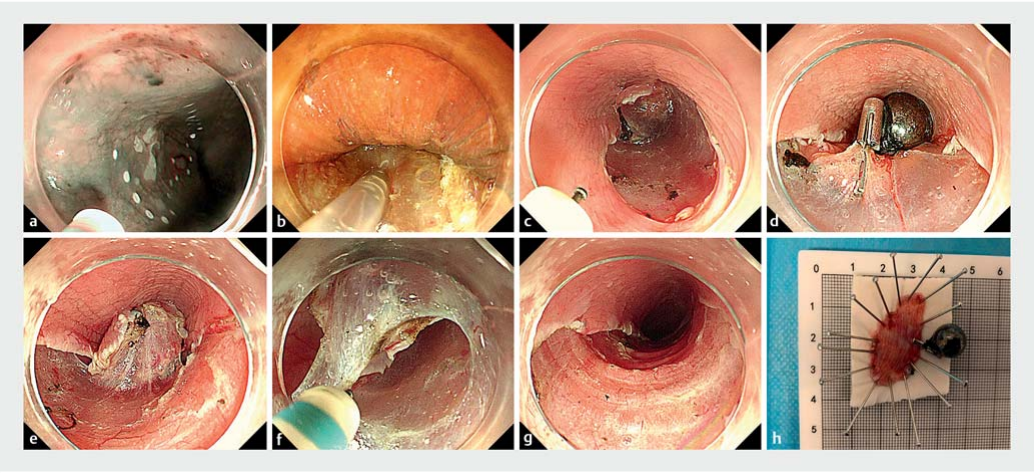
Magnet system-assisted endoscopic submucosal dissection. **a** Mark of the lesion. **b** Submucosal injection. **c** C-shaped mucosal incision. **d** Placement of internal magnetic bead with endoclip. **e** The clearly exposed cutting line with adjustment of the external magnet to approach the internal magnetic bead. **f** Submucosal dissection. **g** Postoperative wound. **h** Specimen and internal magnetic bead.

**Video 1** Magnet-assisted traction method helps to reduce the difficulty of esophageal endoscopic submucosal dissection.


Clip-line traction is commonly used to reduce the technical difficulty of esophageal ESD, but it is not always adequate because it is difficult to adjust the direction of traction if necessary 3 4 . We performed this technique for the first time in esophageal ESD, but we have previous experience in colorectal ESD 5 , and this case demonstrates the safety and effectiveness of this technique in esophageal ESD. However, follow-up studies are needed to further evaluate the technique.

Endoscopy_UCTN_Code_TTT_1AO_2AG

## Notes

Endoscopy E-Videos

https://eref.thieme.de/e-videos
*E-Videos* is an open access online section of the journal *Endoscopy* , reporting on interesting cases and new techniques in gastroenterological endoscopy. All papers include a high-quality video and are published with a Creative Commons CC-BY license. Endoscopy E-Videos qualify for HINARI discounts and waivers and eligibility is automatically checked during the submission process. We grant 100% waivers to articles whose corresponding authors are based in Group A countries and 50% waivers to those who are based in Group B countries as classified by Research4Life (see: https://www.research4life.org/access/eligibility/ ). This section has its own submission website at https://mc.manuscriptcentral.com/e-videos
